# Impact of Paraesophageal Hernia Repair on Respiratory Function: A Systematic Review

**DOI:** 10.3389/fsurg.2021.666686

**Published:** 2021-06-28

**Authors:** Dominick Myers, Xander Jacobson, Matthew Dale, Venket Sahasranaman, Kalyana Nandipati

**Affiliations:** ^1^School of Medicine, Creighton University, Omaha, NE, United States; ^2^Department of Surgery, School of Medicine, Creighton University, Omaha, NE, United States; ^3^Department of Pulmonology, School of Medicine, Creighton University, Omaha, NE, United States

**Keywords:** hernia, gastroesophageal reflux disease, pulmonary function, visceral surgery, lung

## Abstract

**Background and Objectives:** Surgical repair of hiatal and paraesophageal hernia is widely accepted for the treatment of gastroesophageal reflux symptoms. The respiratory benefit of this surgery is less clear. The objective of this review is to quantify the benefit to pulmonary function and subjective dyspnea of paraesophageal hernia repair with the aim of refining the indications and contraindications for elective paraesophageal hernia repair.

**Methods:** Articles were gathered from systematic searches of the Medline Complete Database via the Creighton University Health Sciences Library literature search services. Publications with both pre and postoperative pulmonary function data or both pre and postoperative subjective dyspnea data with regards to surgical paraesophageal hernia repair were included.

**Results:** Six studies were included in this review. The majority of studies in this review show improvement in pulmonary function postoperatively with regards to FEV1, FVC, and VC when stratified by % intrathoracic stomach (ITS), particularly in groups >50% ITS. No significant change was seen in postoperative DLCO or FEV1/FVC.

**Conclusion:** Paraesophageal hernia repair has shown to improve pulmonary function both objectively and subjectively. This review was limited by the paucity of literature on the subject as well as the lack of a standardized method for measurement of %ITS.

## Introduction

Paraesophageal hernias are defined as herniation of the abdominal contents into the thorax through the esophageal hiatus. Paraesophageal hernias are a prevalent condition, cited as being present in 60% of the population over 50 in the United States (1). Pathological manifestations of paraesophageal hernias include the displacement of the gastroesophageal (GE) junction superior to the diaphragm (type I or sliding), herniation of the gastric fundus superior to the diaphragm with the GE junction remaining in place (type II), a combination of GE junction and fundus displacement (type III), and lastly the herniation of additional organs superior to the diaphragm (type IV) (2). Patient presentations vary, and include: remaining asymptomatic, gastroesophageal reflux disease (GERD), early satiety, pain after meals, Cameron's ulcers, iron-deficiency anemia, more rarely, gastric volvulus, and respiratory complications due to compression of the lungs (3). When symptomatic, surgical intervention may be indicated for paraesophageal hernias.

Sliding hiatal hernias, or type 1 paraesophageal hernias, generally are discovered in a workup of GERD. These hernias manifest as gastric tissue moving above and below the diaphragm as a part of the respiratory cycle. This leads to improper gastro-esophageal sphincter pressure and regurgitation of gastric contents, manifesting clinically as GERD, regurgitation, and dysphagia. While this type of hernia can contribute to respiratory issues through chronic micro-aspiration, most studies observed did not include this type of hernia. This review tends to focus on either giant hernias (type II-IV), although one study did separate data based on type of hernia and included type II-IV (2).

The impact of paraesophageal hernias on pulmonary function has been reported by several retrospective studies, by both subjective and objective measures (4). Low and Simchuk reported that pulmonary function test (PFT) results improved after repair of large paraesophageal hernias along with the symptoms like dyspnea (5). However, it is unclear from the current literature whether the size of paraesophageal hernias has any direct correlation with severity of dyspnea and variations in PFTs. It is also unclear whether paraesophageal hernia repair results in improvement in pulmonary function, or just subjective dyspnea (4–6). This systematic review was performed to understand the relation between paraesophageal hernia repair and the subjective and objective improvements in pulmonary function.

## Methods

### Literature Search

An independent literature search was conducted by two different authors (XJ and DM) on February 02, 2020 utilizing the Medline Complete database employing the PRISMA 2020 checklist (Supplementary Table 3, Supplementary Figure 3) (7). Limitations were set at date of publications from 2000 to 2020, English language, and human subjects. Search MeSH terms included “hernia, hernia/SU” and one search term (Supplementary Figure 1). Additionally, a further literature search that included the MeSH terms (“hernia, hernia/SU” and “respiration disorders” or “hernia/hernia/SU” and “Surveys and Questionnaires+”) was performed to broaden the search. These criteria produced a total of 145 articles (search procedure outlined Supplementary Table 1, and Supplementary Figure 3). A registered review protocol was not used, however, the search strategy is outlined here and in the Supplementary Materials.

### Inclusion and Exclusion Criteria

Inclusion criteria included: (1) a prospective or retrospective study that provided respiratory function data in patients undergoing paraesophageal hernia repair, (2) were published in English, (3) included human participants, and (4) reported pre-operative and post-operative respiratory function status (e.g., spirometry or CT scan) or cardiovascular function status (e.g., echocardiography), or (5) pre- and post-operative subjective respiratory symptom reports. Exclusion criteria included: (1) Lack of reported pre- or post-operative data. The protections against bias in each study were evaluated independently by two researchers using the Methodological Index for Non-Randomized Studies (MINORS) (8) for cohort studies. Their assessments are presented in the Supplementary Table 2.

### Types of Participants and Outcome Measures

The patients in the studies were adults undergoing paraesophageal hernia repair, as outlined by the respective publication. Characteristics of the sample population included percent intrathoracic stomach (ITS%), age of the population, and percentage of male and female patients. Primary outcomes in the studies included respiratory function parameters obtained via pulmonary function tests including: forced vital capacity (FVC), forced expiratory volume in one second (FEV_1_), mean forced expiratory flow between 25 and 75% of the FVC (FEF_25−75_), total lung capacity (TLC), vital capacity (VC), residual volume (RV), and diffusing capacity of lung for carbon monoxide (DLCO). Subjective measurements of dyspnea post operatively were also included. Data was extracted by two independent researchers on pre-assembled data templates in Microsoft Excel. Summary measures between studies were not generated and only intra-study values are reported.

## Results

### Literature Search and Exclusion

Utilizing the Creighton University Health Sciences Library literature search services, three literature searches were performed (Supplementary Figure 2). The first focused on paraesophageal hernia repair and pulmonary function tests and returned seven results. Of these, six met the inclusion criteria outlined in the methods section. A second literature search focused on paraesophageal hernia repair and lung disease, this returned 24 results. Of these two met the inclusion criteria, but both were duplicates identified in the first literature search. A third search was performed, focused on paraesophageal hernia repair and subjective symptom questionnaires, and returned 116 results; however, none of these meet inclusion criteria. In total, 145 articles were collected, and six met the inclusion criteria.

## Functional Measurements

### Forced Expiratory Volume in One Second (FEV_1_)

Three studies performed measurements of FEV_1_ stratified by % ITS (Table 1). Low and Simchuk found improvement in FEV_1_ after paraesophageal hernia repair in each category of % ITS sizes >50% (5). Naoum et al. in the initial study, reported an increase in FEV_1_ following paraesophageal hernia repair (*p* = 0.03) (10). Carrott et al. found a FEV_1_ increase across all categories of % ITS in regards to FEV_1_ (*p* < 0.05), with FEV_1_ increasing in a positive correlation with size of paraesophageal hernia (9). Naoum et al. performed a follow up study in 2017, in which they reported similar FEV_1_findings for overall ITS patients. However, when stratified by hernia size, significant difference was only observed in the >75% ITS group (*p* = 0.03) (4).

**Table 1:** Functional lung measurements.

	**Naoum et al. (4) *n =* 45**				**Carrott et al. 9)*n =* 120**				**Naoum et al. (10)***n =30*****				**Low and Simchuk** (**5**)***n =* 45**			
**FEV_**1**_**	***Pre-***	***Post-***	**Δ**	***p-value***	***Pre-***	***Post-***	**Δ**	***p-value***	***Pre-***	***Post-***	**Δ**	***p-value***	***Pre-***	***Post-***	**Δ**	***p-value***
*All* * <50% ITS* *50–75% ITS* *>75% ITS* *100% ITS*	2.06 ± 0.67 2.01 ± 0.62 2.04 ± 0.48 2.12 ± 0.86	2.19 ± 0.66 2.09 ± 0.61 2.20 ± 0.41 2.29 ± 0.86	+0.130 +0.080 +0.160 +0.170	0.003 0.29 0.0540.03			+0.236 +0.083 +0.099 +0.214 +0.362	<0.05 <0.05 <0.05 <0.05 <0.05	2.11	2.18	+0.070	0.03	1.87 2.011 2.181 1.871 1.791	2.17 2.231 2.361 2.151 2.141	+0.300 +0.220 +0.180 +0.280 +0.350	<0.0001 <0.01 <0.001 <0.005
**FVC**	***Pre-***	***Post-***	**Δ**	***p-value***	***Pre-***	***Post-***	**Δ**	***p-value***	***Pre-***	***Post-***	**Δ**	***p-value***	***Pre-***	***Post-***	**Δ**	***p-value***
*All* * <50% ITS* *50–75% ITS* *>75% ITS* *100% ITS*	2.83 ± 0.88 2.74 ± 0.81 2.87 ± 0.79 2.86 ± 1.05	3.07 ± 0.86 2.88 ± 0.84 3.16 ± 0.66 3.17 ± 1.04	+0.240 +0.140 +0.290 +0.310	<0.001 0.15 0.008 0.001			*+0.303* *+0.130* *+0.163* *+0.279 +0.435*	<0.05 <0.05 <0.05 <0.05 <0.05	2.72	2.98	+0.260	0.001	2.52 3.21 2.82 1.43 1.54	2.89 3.26 3.12 2.75 3.04	+0.300 +0.220 +0.180 +0.280 +0.350	<0.0001 <0.02 <0.001 <0.002
**FEV**_**1**_**/FVC**	***Pre-***	***Post-***	**Δ**	***p-value***	***Pre-***	***Post-***	**Δ**	***p-value***	***Pre-***	***Post-***	**Δ**	***p-value***	***Pre-***	***Post-***	**Δ**	***p-value***
*All* * <50% ITS* *50–75% ITS* *>75% ITS* *100% ITS*	73 ± 8 73 ± 8 72 ± 8 74 ± 8	72 ± 6 73 ± 7 70 ± 7 72 ± 6		0.10 0.64 0.27 0.20					72	71		0.05				

*FEV_1_ and FVC values are reported in liters*.

*FEV_1_/FVC is reported as a percent*.

### Forced Vital Capacity (FVC)

Four studies show an improvement in FVC following paraesophageal hernia repair (Table 1). Low and Simchuk found an observed increase in FVC post-operatively overall (*p* <0.0001) and for >50% ITS (5). Naoum et al. observed an overall FVC increase (*p* = 0.001) (10). Carrott et al. observed a significant increase in the FVC across all % ITS categories (*p* <0.05) (9). Naoum et al. observed an increase in FVC overall (*p* <0.001) and for all categories >50% ITS (4).

### FEV_1_/FVC Ratio

Two studies by Naoum et al. calculated the FEV_1_/FVC ratio, with one providing stratification by % ITS (Table 1). A 2011 study by Naoum et al. showed a decrease in the FEV_1_/FVC ratio (*p* <0.05) (10). However, the later report didn't find any significant difference in the ratio of FEV_1_/FVC ratio (*p* = 0.10) (4) in ITS patients after surgical intervention.

## Volume Measurements and Diffusion Capacity

### Total Lung Capacity (TLC)

Total lung capacity data can be found in Table 2. An overall increase in TLC was observed by Naoum et al. in the initial (2011, *p* = 0.03) and subsequent study (2017, *p* = 0.0008) (4, 10). After stratifying for % ITS, Naoum et al. observed an increase in TLC in the category of 50–75% ITS (*p* = 0.002) (4).

**Table 2:** Lung volumes and diffusion capacity.

	**Naoum et al**. **(****4****)** ***n=*** **45**				**Carrott et al**. **(****9****)** ***n=*** **120**				**Naoum et al**. **(****10****)** ***n=*** **30**				**Low and Simchuk** **(****5****)** ***n=*** **45**			
**TLC**	***Pre-***	***Post-***	**Δ**	***p-value***	***Pre-***	***Post-***	**Δ**	***p-value***	***Pre-***	***Post-***	**Δ **	***p-value***	***Pre-***	***Post-***	**Δ**	***p-value***
*All* * <50% ITS* *50-75% ITS* *>75% ITS* *100% ITS*	*n =* 41 4.78 ± 1.09 4.73 ± 0.910.82 ± 1.16 4.80 ± 1.24	*n =* 41 4.99 ± 1.10 4.86 ± 0.99 5.09 ± 1.07 5.02 ± 1.28	+0.210 +0.130 +0.270 +0.220	0.0008 0.24 0.002 0.54					4.52	4.62	+0.100	0.03				
**VC**	***Pre-***	***Post-***	**Δ**	***p-value***	***Pre-***	***Post-***	**Δ**	***p-value***	***Pre-***	***Post-***	**Δ**	***p-value***	***Pre-***	***Post-***	**Δ**	***p-value***
*All* * <50% ITS* *50–75% ITS* *>75% ITS* *100% ITS*	*n =* 41 2.93 ± 0.87 2.94 ± 0.83 2.94 ± 0.75 2.90 ± 1.05	*n =* 41 3.19 ± 0.93 3.08 ± 0.88 3.27 ± 0.76 3.22 ± 1.15	+0.260 +0.140 +0.330 +0.320	<0.0001 0.20 0.009 0.003			**Δ** +0.286 +0.088 +0.148 +0.245 +0.432	<0.05 <0.05 <0.05 <0.05 <0.05								
**RV**	***Pre-***	***Post-***	**Δ**	***p-value***	***Pre-***	***Post-***	**Δ**	***p-value***	***Pre-***	***Post-***	**Δ**	***p-value***	***Pre-***	***Post-***	**Δ**	***p-value***
*All* * <50% ITS* *50–75% ITS* *>75% ITS* *100% ITS*	*n =* 41 1.86 ± 0.38 79 ±0.31 ± 0.51 1.90 ± 0.30	*n =* 41 1.80 ± 0.34 1.78 ± 0.21 1.83 ± 0.44 1.80 ± 0.35	−0.060 −0.010 −0.050 −0.100	0.39 0.94 0.59 0.21					1.85	1.69	-0.160	0.10				
**DLCO**	***Pre-***	***Post-***	**Δ**	***p-value***	***Pre-***	***Post-***	**Δ**	***p-value***	***Pre-***	***Post-***	**Δ**	***p-value***	***Pre-***	***Post-***	**Δ**	***p-value***
*All* * <50% ITS* *50–75% ITS* *>75% ITS* *100% ITS*	*n =* 43 16.20 ± 4.79 16.89 ± 3.89 14.65 ± 3.53 16.87 ± 6.19	*n =* 43 16.39 ± 4.22 17.06 ± 3.31 14.97 ± 3.72 16.96 ± 5.18	+0.190 +0.170 +0.320 +0.090	0.24 0.68 0.44 0.54			+0.582 +0.360 +0.313 +0.445 +0.873	<0.05 <0.05 <0.05 <0.05 <0.05	15.15	15.20	+0.050	0.32	15.36	15.98	+0.620	0.2

*TLC, VC, and RV are reported in liters*.

*DLCO is reported in mL/mmHg/min*.

### Vital Capacity (VC)

Vital capacity data can be found in Table 2. An overall increase in VC post-operatively was observed by Carrott et al. (*p* < 0.05) for all categories of % ITS (9). Naoum et al. observed an overall increase in VC postoperatively (*p* < 0.0001) (4). When stratified by % ITS by Naoum et al. revealed a significant increase for 50–75% ITS (*p* = 0.009) and >75% ITS (*p* = 0.003).

### Residual Volume (RV)

Residual volume data can be found in Table 2. Naoum et al. in 2011 and 2017 didn't observe any significant changes in residual volume overall (*p* = 0.39 and *p* = 0.10) respectively or across % ITS stratification (4, 10).

### Diffusion Capacity of the Lung for Carbon Monoxide (DLCO)

Diffusion capacity of the lung for carbon monoxide data can be found in Table 2. Low and Simchuk (*p* = 0.2), Naoum et al. (*p* = 0.32) and Naoum et al. (*p* = 0.24) found no significant improvement in DLCO postoperatively (4, 5, 10). For Naoum et al. this insignificance was maintained across all categories of % ITS pre- and post-operatively (4). Alternatively, Carrott et al. found an overall increase in DLCO post-operatively (*p* < 0.05), that did not hold up following stratification across % ITS (*p* >0.05) (9).

## Hernia Classification and Dyspnea

### Spirometry Stratified by Paraesophageal Hernia Type Classification

As seen in Table 3, Carrott et al. further stratified spirometry data by classification of paraesophageal hernia (9). There were no changes observed in FEV_1_, FVC, FEF_25−75_, and DLCO based upon the classification of hernia (*p* >0.05) (9). There was significant improvement seen in VC across all categories of paraesophageal hernia (*p* < 0.05) (9).

**Table 3:** Spirometry values stratified by hiatal hernia classification Carrott et al. (9) *n* = 120.

	**FEV**_****1****_		**FCV**		**IsoFEF**_****25−75****_	
	**Δ**	***p-value***	**Δ**	***p-value***	**Δ**	***p-value***
All	+0.236	<0.001	+0.303	<0.001	+0.249	<0.001
Type II *n =* 3	+0.353	>0.05	+0.463	>0.05	+0.413	>0.05
Type III *n =* 92	+0.209	>0.05	+0.268	>0.05	+0.207	>0.05
Type IV *n =* 25	+0.319	>0.05	+0.409	>0.05	+0.386	>0.05
	**VC**		**DLCO**			
	**Δ**	***p-value***	**Δ**	***p-value***		
All	+0.286	<0.001	+0.582	0.004		
Type II *n =* 3	+0.460	>0.05	−0.867	>0.05		
Type III *n =* 92	+0.242	>0.05	+0.505	>0.05		
Type IV *n =* 25	+0.428	>0.05	+1.050	>0.05		

*FEV_1_, FVC, and VC reported in liters*.

*IsoFEF_25−75_ (forced expiratory flow rate between 25 and 75% of FVC) is reported in liters*.

*DLCO is reported in mL/mmHg/min*.

### Pulmonary Function Improvement by Dyspnea Symptomatology

As seen in Table 4, Zhu et al. stratified the pulmonary function test data by the presence of dyspnea, improvement of dyspnea post-repair, and resolution of dyspnea post-repair. This study failed to find a significant changes in any of the groups with regards to FEV_1_, FVC, TLC, RV, and DLCO (6).

**Table 4:** Spirometry values stratified by dyspnea Zhu et al. (6).

	**FEV**_****1****_		**FVC**		**TLC**	
	**Δ**	***p-value***	**Δ**	***p-value***	**Δ**	***p-value***
All	+0.050	0.148	+0.100	0.121	+0.100	0.393
None *n =* 4	+0.050	0.287	+0.100	0.187	+0.100	0.254
Improved *n =* 4	+0.000	0.670	+0.100	0.114	+0.100	0.125
Resolved *n =* 22	+0.070	0.441	+0.080	0.09	+0.200	0.457
	**RV**		**DLCO**			
	**Δ**	***p-value***	**Δ**	***p-value***		
All	−0.100	0.092	+0.600	0.264		
None *n =* 4	+0.000	0.912	−0.900	0.465		
Improved *n =* 4	+0.100	0.147	+0.600	0.439		
Resolved *n =* 22	−0.100	0.314	+0.900	0.537		

*FEV_1_, FVC, TLC, and RV are reported in liters*.

*DLCO is reported in mL/mmHg/min*.

### Qualitative Dyspnea Measures

Shown in Table 5, dyspnea was the independent variable for a study by Li et al. (11) and Zhu et al. (6). Zhu et al. measured for the presence of subjective symptoms, but did not utilize a severity score (6). They found a significant reduction in the presence of heartburn, chest pain, dyspnea, and dysphagia after surgical correction of paraesophageal hernia (6).

**Table 5:** Subjective symptoms related to hiatal hernia repair.

**Hernia characteristics and symptoms duration Li et al**. **(****11****)**							
**Symptom duration vs. Hernia type**				**Symptom duration vs. Hernia size**			
**Hernia Type**	*** <10 years***	***>10 years***	***p-value***	**Hernia Size**	*** <10 years***	***>10 years***	***p-value***
	***n=52***	***n=28***			***n=52***	***n=28***	
Type I	15 (33.3%)	6 (21.4%)	0.472				
Type II	13 (25.0%)	4 (14.3%)	0.264	<5 cm	18 (34.6%)	6 (21.4%)	0.220
Type III	8 (15.4%)	2 (7.1%)	0.288	5–10 cm	14 (26.9%)	4 (14.3%)	0.197
Type IV	16 (30.8%)	16 (57.1%)	0.022	>10 cm	20 (38.5%)	18 (64.3%)	0.027
**Pre- and Post-operative changes in symptom scores Li et al**. **(****11****)**									
	**GERD patients**				**Hiatal hernia patients**				**GERD vs. HH**
**Symptoms**	***Pre- (n=56)***	***Post- (n=54)***	**Δ**	***p-value***	***Pre- (n=80)***	***Post- (n=76)***	**Δ**	***p-value***	***P-value***
Heartburn	4.9 ± 1.4	2.5 ± 1.8	−2.4	<0.0001	5.6 ± 1.4	1.6 ± 1.4	−4.0	<0.0001	0.001
Regurgitation	5.2 ± 1.7	2.4 ± 1.8	−2.8	<0.0001	5.8 ± 1.4	1.6 ± 1.2	−4.2	<0.0001	0.005
Cough	3.5 ± 1.9	2.4 ± 1.7	−1.1	0.003	4.2 ± 1.7	1.8 ± 1.2	−2.4	<0.0001	0.018
Wheezing	3.8 ± 1.3	2.4 ± 1.7	−1.4	<0.0001	4.5 ± 1.4	1.8 ± 1.3	−2.7	<0.0001	0.016
Chest pain	2.9 ± 1.7	1.6 ± 1.4	−1.3	0.023	3.6 ± 1.5	1.6 ± 1.4	−2.0	<0.0001	0.020
**Pre- and Post-operative changes in symptom outcomes Zhu et al**. **(****6****)** **(*****n*** **=** **30)**							
	**Number**			**Severity index mean (scale max of 4)**			
**Symptoms**	***Pre-***	***Post-***	**Δ**	***Pre-***	***Post-***	**Δ**	***P-value***
Heartburn	18 (60%)	5 (16%)	−13	2.2	1.3	−0.9	0.003
Regurgitatios	10 (33%)	1 (3%)	−9	1.7	1.0	−0.7	0.005
Chest Pain	16 (53%)	2 (3%)	−14	2.0	1.1	−0.9	<0.001
Dysphagia	15 (50%)	2 (7%)	−13	2.1	1.1	−1.0	0.001
Dyspnea	26 (87%)	4 (13%)	−22	2.4	1.3	−1.1	<0.001

Li et al. measured subjective improvement of pre-operative symptoms based upon a subjective symptom index score (11). They found a significant improvement in subjective heartburn, regurgitation, cough, wheezing, and chest pain after paraesophageal hernia correction (11).

## Discussion

The current systematic review explored the relation between pulmonary function and paraesophageal hernia repair. One mechanism involves reflux related worsening of asthma and tissue level damage. Another relates to pulmonary compression from herniated abdominal contents. One objective of this review is to identify which mechanism plays a larger role in dyspnea production in the studies surveyed. We identified five studies which met our criteria and reported pre- and post-operative pulmonary function objectively, while one additional study included only subjective data.

Patient selection for paraesophageal hernia repair is designed to treat those with GERD refractory to medical management or other gastroesophageal complications and not generally provide relief from respiratory symptoms due to possible lung compression. This strategy is supported by an only modest observed improvement in pulmonary function tests after hernia repair. Objectively, there was significant post-operative increase in TLC and VC, with no significant alteration in the RV (4, 9). Improvements in TLC and VC were proposed secondary to the physical removal of gastric tissue from the thoracic cavity. One would predict further increases in these lung volumes relative to the total thoracic volume occupied, as reflected by the ITS. Reduced TLC and VC are characteristic of a restrictive lung picture, diagnosed by <5th percentile of predicted for both TLC and VC (12). Significant improvement in both these volumes suggested a restrictive lung pathology (12). This further corroborated by a lack of RV alterations in these studies. This observation implies the presence of intrathoracic gastric tissue potentially reduces the functional capacity of the lungs and is further supported by PEH repair patients having significant improvements in TLC and VC postoperatively.

Differences were observed between the ITS categories, with an increase in post-operative TLC in the overall group and patients with ITS 50–75%. There was no significant improvement seen in TLC for patients with <50% ITS and >75% (4). While TLC and VC at baseline correlated with % ITS, improvement in these volumes is not as clear. This may be attributed to irreversible changes to TLC, such as confounding underlying disease, that occur at higher % ITS; however, further research is necessary given the small sample examining the TLC and VC relationship. Similarly to TLC, Naoum et al. found a significant increase in VC in categories of patients with ITS >50%, not observed in the patient sample with ITS <50% (4). While Carrott et al. observed a significant increase in post-operative VC across all categories of ITS (9).

The FVC and FEV_1_ are other objective pulmonary functions which showed significant improvement in the majority of studies (4, 5, 9). Improvement of the FVC and FEV_1_ theoretically represent the relief of both airway obstruction due to gastric compression of respiratory outlets and decreased restriction due to increased volume available for lung expansion (4). However, the extent to which airway obstruction contributes to diminished pulmonary function is minimal; supported by Naoum et al. finding no significant improvement in the post-operative FEV1/FVC ratio and identifying no change in airway resistance post-operatively (4). Additionally, there was no correlation in the FEV_1_/FVC ratio with the % ITS, further supporting this assertion (4). Carrott et al. found an increase in FEF_25−75_ following repair (4, 9). Improvement across these spirometry values further suggests that the increase in TLC and VC increases the functional ability of the lungs. Study parameters such as FEV_1_/FVC ratio and DLCO are often used to assess restrictive vs. obstructive lung disease. With a normal baseline FEV_1_/FVC ratio in combination with improved TLC and VC, the limitation on pulmonary function follows a restrictive pattern (4). No post-operative change in DLCO suggests, expectedly, the source of respiratory concerns due to paraesophageal hernias is not occurring at the lung tissue level, such as would be seen with pulmonary fibrosis, but rather the effects of paraesophageal hernias are most likely due to the reduction in thoracic volume (4–6, 9). This data seems to suggest an improvement primarily due to correction of space occupying gastric tissue, rather than micro-aspiration causing lung tissue damage.

Improvement was found to be dependent upon the degree of ITS experienced by the patient. Naoum et al. and Low and Simchuk observed an increase in both FVC and FEV_1_ in patients afflicted with >50% ITS, but not in those with <50% ITS (4, 5). Alternatively, Carrott et al. observed improvement across all categories (9). Estimation of successful improvement in objective pulmonary function, therefore, can be predicted based upon the percentage of ITS a patient presents with upon surgical intervention, with higher rates of improvement for those with >50% ITS. The type of paraesophageal hernia afflicting a patient has very little impact on pulmonary function test outcome (9).

While recovery of objective pulmonary function is observed, this does not necessarily translate to clinical practice. Assessing for subjective improvement in dyspnea, Zhu et al. grouped their study based upon the presence or lack of subjective improvement in dyspnea postoperatively (6). Despite an overall improvement in dyspnea reported by the sample, no objective significant difference was noted in respiratory function between the groups (6). This was found across the following parameters: FVC, FEV_1_, TLC, RV, and DLCO (6).

Common symptoms experienced by patients afflicted with paraesophageal hernia include, heartburn, early satiety, chest pain, regurgitation, dysphagia, anemia, more rare symptoms include dyspnea and wheezing (5, 6, 9, 11). Prior to surgical correction, the size of hernia is correlated with the length of GERD symptoms, with >10 cm paraesophageal hernia is a significant risk factor in GERD symptoms persisting for >10 years (11). Paraesophageal hernia repair is effective in alleviating, to some degree: dyspnea, heartburn, chest pain, regurgitation, cough, wheezing, and dysphagia (6, 11). Regarding patients with GERD and paraesophageal hernia, improvement in dyspnea symptoms (cough and wheezing) was significantly greater than the improvement seen in those with GERD alone. However, both groups saw significant improvement over preoperative baseline (11).

The morbidity of paraesophageal hernia repair can be 3–45% of patients and it is important to properly select patients where improvement is expected (3). Additionally, it is vital to select patients in which the risk of surgery does not outweigh the potential benefits; pulmonary complications and congestive heart failure in a pre-operative setting is predictive of increased post-operative mortality risk (13). In identifying patients who will be positively impacted, clinicians should focus on patients experiencing subjective respiratory (e.g., dyspnea, cough, wheeze) and gastrointestinal (e.g., refractory GERD, chest pain, dysphagia) symptoms. As suggested by the results of this systematic literature review, priority should be given to patients with greater ITS fraction, specifically those with >50% ITS.

Limitations of this review include the small literature sample, with only six included results, it is an understudied topic in our academic setting. Lack of consistent reporting of demographic data and pre-operative comorbid conditions within several studies limits generalizability. The measurement of ITS is often assessed by a surgeon based upon anatomical relationships of the hernia and gastric components observed in. There is no standardized method for classification of ITS intra-operatively, which limits the comparisons between surgeons. More widespread use and reporting of radiographic swallow studies could provide more concrete data on hernia size. Further research should be directed toward a standardized protocol for evaluating %ITS intraoperatively. The studies included did not include data for patients with a <50% ITS, as studies on this subgroup did not include pulmonary data or did not include surgical intervention. The effects of small paraesophageal hernias on pulmonary function may be different than those described in this review. Studies included did not follow patients for improvement in refluxate correlated with pulmonary function tests long term. A study of this type would provide stronger evidence for the precise mechanism of improvement.

## Data Availability Statement

The original contributions presented in the study are included in the article/Supplementary Material, further inquiries can be directed to the corresponding author/s.

## Author Contributions

DM, XJ, KN, MD, and VS contributed to conception and design of the study. DM and XJ performed the literature search and wrote the first draft of the manuscript. All authors contributed to manuscript revision, read, and approved the submitted version.

## Conflict of Interest

The authors declare that the research was conducted in the absence of any commercial or financial relationships that could be construed as a potential conflict of interest.
