# Supplementary material for: Impact of Paraesophageal Hernia Repair on Respiratory Function: A Systematic Review
Source: Front Surg. 2021 Jun 28;8:666686. doi: 10.3389/fsurg.2021.666686 (PMC8273160; doi:10.3389/fsurg.2021.666686)
Supplement: Supplementary file 1 [file Data_Sheet_1.docx]

| **Search Number** | **Query**  **Supplemental Table 1.** | **Limiters and Expanders** | **Last Run Via** | **Results Returned** |
| --- | --- | --- | --- | --- |
| S7 | S1 AND S6 | Expanders - Apply equivalent subjects Search modes - Boolean/Phrase | Interface - EBSCOhost Research Databases Search Screen - Advanced Search Database - MEDLINE Complete | 116 |
| S6 | (MH "Surveys and Questionnaires+") | Expanders - Apply equivalent subjects Search modes - Boolean/Phrase | Interface - EBSCOhost Research Databases Search Screen - Advanced Search Database - MEDLINE Complete | 998,698 |
| S5 | S1 AND S4 | Expanders - Apply equivalent subjects Search modes - Boolean/Phrase | Interface - EBSCOhost Research Databases Search Screen - Advanced Search Database - MEDLINE Complete | 24 |
| S4 | (MH "Respiration Disorders+") | Expanders - Apply equivalent subjects Search modes - Boolean/Phrase | Interface - EBSCOhost Research Databases Search Screen - Advanced Search Database - MEDLINE Complete | 186,606 |
| S3 | S1 AND S2 | Expanders - Apply equivalent subjects Search modes - Boolean/Phrase | Interface - EBSCOhost Research Databases Search Screen - Advanced Search Database - MEDLINE Complete | 7 |
| S2 | (MH "Respiratory Function Tests+") OR (MH "Valsalva Maneuver") OR (MH "Work of Breathing") OR (MH "Ventilation- Perfusion Scan") OR (MH "Spirometry") OR (MH "Bronchospirometry") OR (MH "Pulmonary Ventilation") OR (MH "Maximal Voluntary Ventilation") OR (MH "Forced Expiratory Volume") OR (MH "Forced Expiratory Flow Rates") OR (MH "Peak Expiratory Flow Rate") OR (MH "Maximal Midexpiratory Flow Rate") OR (MH "Maximal Expiratory Flow- Volume Curves") OR (MH "Maximal Expiratory Flow Rate") OR (MH "Pulmonary Gas Exchange") OR (MH "Ventilation-Perfusion Ratio") OR (MH "Pulmonary Diffusing Capacity") OR (MH "Plethysmography, Whole Body") OR (MH "Maximal Respiratory Pressures") OR (MH "Lung Volume Measurements") OR (MH "Total Lung Capacity") OR (MH "Vital Capacity") OR (MH "Inspiratory Capacity") OR (MH "Tidal Volume") OR (MH "Inspiratory Reserve Volume") OR (MH "Expiratory Reserve Volume") OR (MH "Functional Residual Capacity") OR (MH "Residual Volume") OR (MH "Closing Volume") OR (MH "Lung Compliance") OR (MH "Exercise Test") OR (MH "Capnography") OR (MH "Bronchial Provocation Tests") OR (MH "Blood Gas Analysis") OR (MH "Oximetry") OR (MH "Blood Gas Monitoring, Transcutaneous") OR (MH "Airway Resistance") OR (MH "Respiratory Mechanics") OR (MH "Inhalation") OR (MH "Exhalation") OR (MH "Bronchoconstriction") OR (MH "Breath Holding") OR (MH "Respiratory Rate") OR (MH "Respiration") OR (MH "Respiratory Transport") OR (MH "Respiratory Physiological Phenomena") | Expanders - Apply equivalent subjects Search modes - Boolean/Phrase | Interface - EBSCOhost Research Databases Search Screen - Advanced Search Database - MEDLINE Complete | 309,153 |
| S1 | (MH "Hernia, Hiatal/SU") | Limiters - Date of Publication: 20000101- 20201231; English Language; Human Expanders - Apply equivalent subjects Search modes - Boolean/Phrase | Interface - EBSCOhost Research Databases Search Screen - Advanced Search Database - MEDLINE Complete | 1,057 |

Search strategy performed by Creighton University Health Sciences Library on the Medline database. This search was performed Sundary February 02, 2020 at 8:12:58 PM.

**Supplemental Figure 1.** Literature Search Terms

| **Pulmonary Function Literature Search Terms** | |
| --- | --- |
| Respiratory function tests  Ventilation-perfusion scan  Bronchospirometry  Maximal voluntary ventilation  Forced expiratory flow rates  Maximal midexpiratory flow rate  Maximal expiratory flow rate  Ventilation-perfusion ratio  Plethysmography, whole body  Lung volume measurements  Vital capacity  Inspiratory reserve volume  Functional residual capacity  Closing volume  Exercise test  Bronchial provocation tests  Oximetry  Airway resistance  Inhalation  Bronchoconstriction  Respiratory rate  Respiratory transport | Valsalva maneuver  Spirometry  Pulmonary ventilation  Forced expiratory volume  Peak expiratory flow rate  Maximal expiratory flow-volume curves  Pulmonary gas exchange  Pulmonary diffusing capacity  Maximal respiratory pressures  Total lung capacity  Tidal volume  Expiratory reserve volume  Residual volume  Lung compliance  Capnography  Blood gas analysis  Blood gas monitoring  Respiratory mechanics  Exhalation  Breath holding  Respiration  Respiratory physiological phenomena |

**
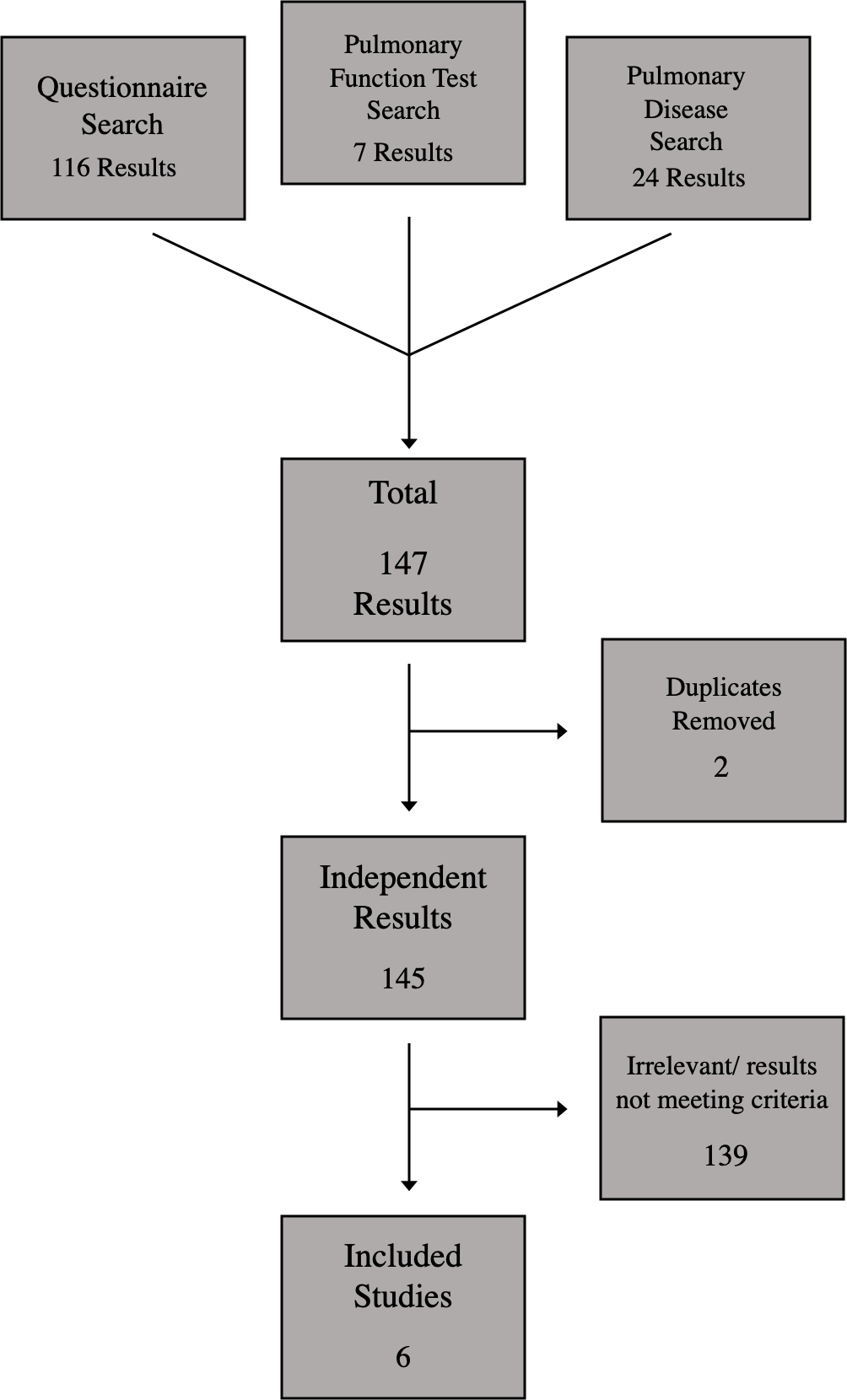
Supplemental Figure 2.** Literature Search Results Flowchart

| **MINORS Bias Assessment Scores** | | | | | | | | | | | | |
| --- | --- | --- | --- | --- | --- | --- | --- | --- | --- | --- | --- | --- |
| Question # | Naoum et al. (2017) | | Carrott et al. (2012) | | Naoum et al. (2011) | | Low & Simchuk (2002) | | Zhu et al. (2011) | | Li et al. (2018) | |
| **Reviewer** | **1** | **2** | **1** | **2** | **1** | **2** | **1** | **2** | **1** | **2** | **1** | **2** |
| 1 | 2 | 2 | 2 | 2 | 2 | 2 | 2 | 2 | 2 | 2 | 2 | 2 |
| 2 | 2 | 2 | 2 | 2 | 2 | 2 | 1 | 1 | 1 | 2 | 2 | 2 |
| 3 | 2 | 2 | 2 | 2 | 2 | 2 | 1 | 1 | 2 | 2 | 0 | 0 |
| 4 | 2 | 2 | 2 | 2 | 2 | 2 | 2 | 2 | 2 | 2 | 2 | 2 |
| 5 | 0 | 0 | 0 | 0 | 2 | 2 | 0 | 0 | 0 | 0 | 0 | 0 |
| 6 | 1 | 2 | 2 | 0 | 2 | 2 | 2 | 2 | 2 | 2 | 2 | 2 |
| 7 | 2 | 2 | 1 | 2 | 1 | 1 | 2 | 2 | 2 | 2 | 1 | 2 |
| 8  9  10  11  12 | 0  1  1  2  2 | 0  2  2  2  2 | 0  1  1  2  2 | 0  2  2  2  2 | 0  1  1  2  2 | 0  2  2  2  2 | 0  1  1  2  2 | 0  2  2  2  2 | 0  1  1  2  2 | 0  2  2  2  2 | 0  1  1  2  2 | 0  2  2  2  2 |
| **Total** | **17** | **20** | **17** | **18** | **19** | **21** | **16** | **18** | **17** | **20** | **15** | **18** |

Scores are out of 2 per question for a total of 24. A score of 2 indicates adequate, 1 indicates present but inadequate, 0 indicates not present. Reviewers scored each study independently and blinded from the other.

**Supplemental Table 2**
