# Supplementary material for: Impact of Paraesophageal Hernia Repair on Respiratory Function: A Systematic Review
Source: Front Surg. 2021 Jun 28;8:666686. doi: 10.3389/fsurg.2021.666686 (PMC8273160; doi:10.3389/fsurg.2021.666686)
Supplement: Supplementary file 2 [file Data_Sheet_2.docx]

**Identification of studies via databases and registers**

Records removed *before screening*:

Duplicate records removed (n =2 )

Records marked as ineligible by automation tools (n =0)

Records removed for other reasons (n =0)

Records identified from*:

Databases (n =1 (Medline)

Registers (n =0 )

**Identification**

Records screened

(n =145 )

Records excluded**

(n = 139)

Reports sought for retrieval

(n =8 )

Reports not retrieved

(n =0 )

**Screening**

Reports assessed for eligibility

(n =8 )

Reports excluded:

Reaseon: Neither pulmonary function or qualitative data included. (n =2)

Studies included in review

(n = 6)

Reports of included studies

(n = 0)

**Included**
